# Neurofeedback Training Based on Motor Imagery Strategies Increases EEG Complexity in Elderly Population

**DOI:** 10.3390/e23121574

**Published:** 2021-11-25

**Authors:** Diego Marcos-Martínez, Víctor Martínez-Cagigal, Eduardo Santamaría-Vázquez, Sergio Pérez-Velasco, Roberto Hornero

**Affiliations:** 1 Biomedical Engineering Group, E.T.S.I. Telecomunicación, University of Valladolid, Paseo de Belén 15, 47011 Valladolid, Spain; victor.martinez@gib.tel.uva.es (V.M.-C.); eduardo.santamaria@gib.tel.uva.es (E.S.-V.); sergio.perez@gib.tel.uva.es (S.P.-V.); robhor@tel.uva.es (R.H.); 2 Centro de Investigación Biomédica en Red en Bioingeniería, Biomateriales y Nanomedicina (CIBER-BBN), 28029 Madrid, Spain

**Keywords:** neurofeedback training (NFT), motor imagery (MI), sample entropy, multiscale entropy (MSE), brain–computer interfaces (BCI), elderly people, age-relate cognitive decline, Luria adult neuropsychological diagnosis (Luria-AND)

## Abstract

Neurofeedback training (NFT) has shown promising results in recent years as a tool to address the effects of age-related cognitive decline in the elderly. Since previous studies have linked reduced complexity of electroencephalography (EEG) signal to the process of cognitive decline, we propose the use of non-linear methods to characterise changes in EEG complexity induced by NFT. In this study, we analyse the pre- and post-training EEG from 11 elderly subjects who performed an NFT based on motor imagery (MI–NFT). Spectral changes were studied using relative power (RP) from classical frequency bands (delta, theta, alpha, and beta), whilst multiscale entropy (MSE) was applied to assess EEG-induced complexity changes. Furthermore, we analysed the subject’s scores from Luria tests performed before and after MI–NFT. We found that MI–NFT induced a power shift towards rapid frequencies, as well as an increase of EEG complexity in all channels, except for C3. These improvements were most evident in frontal channels. Moreover, results from cognitive tests showed significant enhancement in intellectual and memory functions. Therefore, our findings suggest the usefulness of MI–NFT to improve cognitive functions in the elderly and encourage future studies to use MSE as a metric to characterise EEG changes induced by MI–NFT.

## 1. Introduction

Electroencephalography (EEG) is a non-invasive and portable method of monitoring brain activity. This technique is based on recording the electrical activity from pyramidal neurons of the cortex by placing a set of electrodes on the subject’s scalp [1]. Task-related EEG patterns (e.g., visual stimuli or motor intentions) are used by brain–computer interfaces (BCI) to predict the user’s intentions and convert them into commands to control an external device, without using muscles or peripheral nerves [1,2]. Through this direct communication between the subject’s brain and an external device, BCI applications aim to improve the quality of life of people with motor or cognitive disabilities [1,2]. Nevertheless, assisting the disabled is not the only objective of BCI, but also the rehabilitation or recovery of their motor and cognitive functions [3,4].

Neurofeedback training (NFT) is a therapy based on the hypothesis that, due to brain plasticity, effects of neural disorders can be counteracted by inducing the appropriate brain modulation that normalizes the patient’s deviant brain signal [5,6]. In this regard, NFT users are encouraged to modulate their EEG signals (e.g., the power of a specific frequency band or a ratio between band powers), which is expected to have beneficial effects on their brain state and can lead to brain microstructural changes after training [7]. To this end, BCI applications are employed to measure users’ EEG signals and then provide a feedback stimulus that assists them in finding strategies to gain control of their brain signal modulation. Hereafter, we will refer to this training paradigm as classical NFT. In recent years, several studies have investigated NFT-induced neurological improvements in patients with attention-deficit/hyperactivity disorder [8,9] or epilepsy [10], among others. Moreover, age-related brain changes have been shown to lead a power shift from rapid to slow rhythms in brain activity [11,12,13,14]. Thus, NFT has been proposed as a promising strategy to prevent the progression of the effects of age-related cognitive decline [15]. Accordingly, NFT aims to normalize the deviated power spectral distribution of the patient in order to enhance their cognitive functions [5]. In view of increasing life expectancy [16], NFT could improve the social well-being of the growing elderly population in the future. Previous classical NFT studies on healthy young adults showed significant changes in theta (4–8 Hz) [17,18], alpha (8–13 Hz) [19,20,21], and beta (13–30 Hz) [21] band powers. These studies also reported significant improvements in the results of neuropsychological tests for the assessment of memory-related [17,19,21], attention [17,21], and visuospatial functions [20]. Furthermore, several studies have been conducted on elderly subjects [15]. Such studies reported significant differences in the theta [17,22,23], alpha [22,23,24], beta [22,25], and gamma (>30 Hz) [25] band powers, as well as significant improvements in memory-related functions [17,22,23,24,26] and attention [17,22,23,24,26] after the NFT.

On the other hand, EEG activity involved in mental motor imagery (MI) tasks is associated with motor and cognitive functions [27,28]. These MI tasks are based on the mental imagination of a movement without any peripheral muscle activation and produce desynchronization and synchronization events in alpha and beta frequency bands over the contralateral sensorimotor areas [29]. These events are called sensorimotor rhythms (SMR) and can be used as control signals by BCI applications. Hence, some studies have proposed the use of a MI-based NFT paradigm (MI–NFT), instead of classical NFT, as a promising approach to achieve the desired modulation [3,4,30]. The MI–NFT paradigm is broadly extended among studies focusing on neurorehabilitation of post-stroke patients [3,4,31,32,33,34,35]. This paradigm has proven to be effective in promoting functional and structural brain plasticity and recovery of motor function [36]. Furthermore, in contrast to the classical NFT, the MI–NFT paradigm allows to develop BCI applications with two degrees of freedom (right hand vs. left hand MI). Therefore, the developed training interfaces can be more complex. In this sense, studies have suggested that more gamified applications may be beneficial for therapy outcomes [32,37]. In view of the results achieved by the MI–NFT paradigm in neurorehabilitation studies of stroke patients, it is interesting to consider this paradigm for cognitive training. Indeed, gaining control of SMR modulation may be beneficial for patients, as previous research has suggested an active role of alpha brain activity in cognitive functions, such as memory-related processes [38,39,40], intelligence [41], executive functions [40], and attention-related functions [19,39,42], as well as a functional role of beta oscillations in working memory [43,44], language comprehension [39,45], and attention-related functions [19,44,46]. In particular, our previous work reported significant EEG changes in alpha and beta bands, as well as an enhancement of visuospatial, language, memory, and intellectual functions in healthy elderly people after 5 MI–NFT sessions [30]. Despite being a suitable approach to NFT-based cognitive training, there is a lack of research on the impact of the MI–NFT paradigm on users’ brain activity and cognitive functions.

Even though the results from NFT-based cognitive training studies are encouraging, as they reveal older people’s ability to control their own brain activity [15], further analysis of NFT’s effects on the subject’s brain activity is lacking. That is, EEG changes induced by NFT are mainly assessed by linear techniques based on spectral analysis. This may not be enough to determine the overall impact that NFT can produce on the subjects’ brain activity. In this regard, non-linear analysis methods have been proposed as a suitable tool to give insight into brain dynamics due to its non-linear coupling between neuronal populations [12]. Particularly, multiscale entropy (MSE) was proposed to analyse the complexity of biological signals on multiple time scales [47]. This metric has proven useful for exploring changes in EEG complexity [48,49,50,51], which has been shown to be related to cognition state [12,13]. Indeed, a recent study applied for the first time a variation of MSE algorithm to assess the effects of classical NFT on the subject’s EEG complexity, showing higher complexity values after training [52]. These results encourage further study of the NFT’s influence on brain signal complexity.

To the best of our knowledge, changes in EEG complexity induced by MI–NFT in the elderly have not yet been explored. In this sense, we hypothesize that the effects of cognitive training based on the MI–NFT paradigm could also manifest themselves as changes in the complexity of subject’s EEG signal. Thus, differences in complexity between pre- and post-training measures could be expected to be found. To assess these changes, we propose to apply MSE based on sample entropy (*SampEn*) due to its effectiveness in analysing the irregularity and complexity of biological time-series [53]. Therefore, the objective of the present study is two-fold: (*i*) to comprehensively evaluate EEG changes induced in elderly subjects after an MI–NFT and (*ii*) to analyse the cognitive improvement achieved by the subjects after performing the MI–NFT.

## 2. Materials and Methods

### 2.1. Experimental Protocol

**Figure 1:**
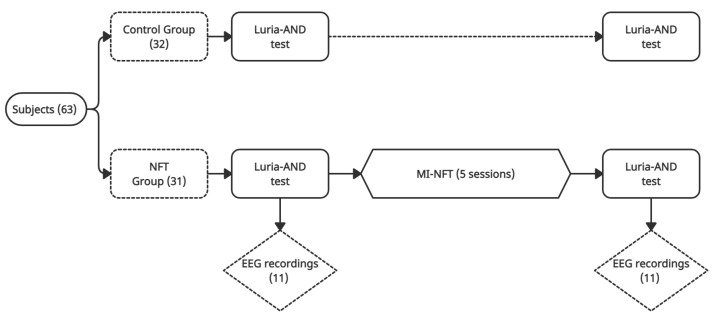
Scheme of the experimental protocol of the MI-based NFT study. An example of the feedback from a MI task is shown.

To assess the changes in brain activity induced by a cognitive training based on MI–NFT, we employed a dataset recorded in our previous work [30]. A scheme of the experimental protocol is shown in Figure 1. The study involved 63 subjects over 60 years recruited from ’Centro de Referencia Estatal de San Andrés del Rabanedo’ (León, Spain). All subjects were healthy, free of psychotropic medication, and without previous psychiatric or neurological disorders or substance abuse. None of them had previous experience in using BCI. They were randomly divided into a control group (32 subjects) and an NFT group (31 subjects). Participants from both groups carried out a Luria adult neuropsychological diagnosis (Luria-AND) test [54] to analyse their neuropsychological status prior to NFT.

In order to perform the training, a novel MI-based BCI tool was developed. Eight active electrodes (F3, F4, T7, C3, Cz, C4, T8, and Pz) were used, placed in an elastic cap according to the international 10-20 system distribution [55]. Ground electrode was located at AFz channel, whilst the common reference was placed in the earlobe. Training tasks were carried out using a g.USBamp amplifier (Guger Technologies OG, Graz, Austria). EEG signals were acquired at 256 Hz sampling rate and processed in real time using the BCI2000 general-purpose system [56]. The feedback stimulus was provided using the band power of tree spectral bands centred on 12, 18, and 21 Hz, with bandwidth of 3 Hz.

The training was performed only by NFT group and consisted of 5 sessions, with an average duration of 90 min over 5 weeks [30]. Five MI tasks with a different level of difficulty were designed. The MI–NFT paradigm allowed the development of tasks with two degrees of freedom which combine the modulation of SMR and the use of cognitive functions:

- Task 1: Subjects were required to imagine hand movements. Alternatively, a closed door or a closed window was displayed on the screen. Then, the paradigm indicated whether users had to perform right- or left-hand MI, respectively. If the user correctly performed the exercise, the displayed object was opened. This task aimed to help subjects learn to modulate their SMR.
- Task 2: A target randomly located on the right or the left of the screen was displayed 3 s prior to the start of the trial. In order to reach the target with a displayed cursor, subjects had to continuously perform MI for a maximum duration of 10 s. The target and the cursor were represented by different pairs of related pictures, such as fish/fridge or person/house.
- Task 3: Subjects had to control a cursor in order to reach one of the two displayed targets (one of them was a picture related to cursor’s picture, and the other was unrelated to it) by performing MI. Targets were displayed 3 s prior to the start of the trial, and subjects had to complete the task in a maximum time of 10 s. Therefore, this task not only involves MI but also the use of logic.
- Task 4: A person walking forward continuously on a path was displayed. Through the MI of their hands, subjects had to control the horizontal position in order to overcome the different obstacles that appeared on the path. This task required the use of the visuospatial function of the subjects. In each trial, the duration of the feedback period was 18 s.
- Task 5: Two pairs of images were shown sequentially for 3 s each, so that one image was common to both pairs. Subjects had to identify the image shown twice and move the cursor towards it by performing MI. They had a maximum of 12 s to do so. Thus, this activity also involved the use of memory.

**Figure 2:**
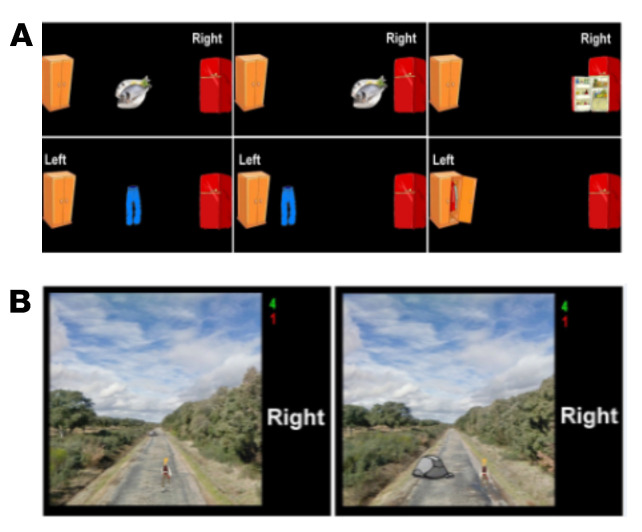
Examples of training interface. Picture (**A**) shows sequences of screenshots from Task 3. In the upper sequence, the cursor is displayed as fish, so the subject has to perform right hand MI to reach the correct target (fridge on the right), while in the lower sequence, the cursor is displayed as trousers, so the subject has to perform left hand MI to reach the correct target (cupboard on the left). In picture (**B**), an example of Task 4 is depicted. The subject has to perform MI in order to overcome the displayed obstacles in real time.

The easiest tasks (i.e., Task 1 and Task 2) were performed at first and the difficulty was increased gradually throughout the sessions. Examples of Task 3 and Task 4 are shown in Figure 2. All subjects performed again the Luria-AND test after the training period to assess possible changes in the different neuropsychological functions. A complete description of the experimental protocol and EEG recording procedures can be found in [30].

### 2.2. Dataset

From the NFT group, pre- and post-training EEG recordings were performed in only 11 subjects (7 females; mean age = 69.4 ± 5.75 years). Therefore, the dataset employed in this study is composed of EEG signals from these subjects. The recordings consisted of two-minute EEG signals in resting state with eyes closed at the beginning of the first session and at the end of the last training session. Detailed socio-demographic information about the 11 NFT subjects under study is shown in Table 1. In addition, the Luria-AND scores of these 11 subjects were used in order to assess their cognitive changes after MI–NFT. A comprehensive analysis of Luria-AND scores of the remaining subjects can be found in [30].

### 2.3. Luria Adult Neuropsychological Diagnosis

Luria-AND [54] is composed of nine tests aimed at covering five different cognitive functions: attention (attentional control test), intellectual (thematic draws and conceptual activity test), memory (immediate memory and logical memory tests), oral language (receptive speech and expressive speech tests), and visuospatial (visual perception and spatial orientation tests) functions. Subjects under study performed the Luria-AND test at the beginning and at the end of the protocol.

### 2.4. Signal Pre-Processing

A notch filter at 50 Hz was previously applied to the data to suppress power line noise. DC component and high frequencies were reduced with a band-pass finite impulse response (FIR) filter, employing a Hamming window in the frequency range 0.1–60 Hz. The filtered EEG was divided into epochs of 10 s each, without overlapping. In this way, visual inspection for artefacts could be carried out. After concluding the inspection of all recordings, no epochs were found that had to be rejected due to the presence of artefacts. This may be due to the fact that, during the 2 min EEG recording, users were asked to avoid eye movement or jaw clenching, which could introduce noise into the signal.

### 2.5. Metrics for Assessing EEG Changes

In order to comprehensively assess EEG changes induced by an MI–NFT, both linear (relative power) and a non-linear (MSE) analyses were applied.

#### 2.5.1. Relative Power

The RP measurement gives information on the normalized weight of each band in the spectral distribution. The spectral bands selected to assess the induced EEG changes were delta (0.1–4 Hz), theta (4–8 Hz), alpha (8–13 Hz), and beta (13–30 Hz). The lower ($lf_{i}$) and upper ($uf_{i}$) frequency limits of each band were specified according to the classical definitions of EEG bands [57]. The power spectral density (PSD) of the signal was calculated by means of the Welch method [58]. A Hamming window of 16 s (4096 samples; spectral resolution of 0.0625 Hz), along with a 90% of overlap and fast Fourier transform of 4096 points, was used. From this magnitude, the relative power (RP) of each band was estimated as follows:

(1) $$RP_{i}\;=\;\frac{\sum {}_{f\;=\;lf_{i}}^{f\;=\;uf_{i}}\mathrm{PSD}\left(f\right)}{\sum {}_{f\;=\;0.1\mathrm{Hz}}^{f\;=\;60\mathrm{Hz}}\mathrm{PSD}\left(f\right)},\;i\;=\;\left\{Delta,Theta,Alpha,Beta\right\}$$

#### 2.5.2. Multiscale Entropy

Traditional non-linear techniques, such as those based on entropy metrics, assess the irregularity of a time series in terms of the presence of repeating patterns [53]. Therefore, the highest values are assigned to random signals. Since complexity analysis methods should reflect the dynamic richness of a system, these measures must assign lowest values to both fully deterministic and fully random time series [53]. According to Costa et al. [53], biological systems need to operate across multiple spatial and temporal scales, which is also reflected in the complexity of their signals across scales. In this context, MSE is a measurement of complexity that fulfils the aforementioned requirement [47,59] and focuses on quantifying the information expressed by the physiologic dynamics over multiple time scales [53]. This is accomplished through estimation of the entropy (i.e., irregularity) on coarse-grained versions of the original signal. As a result of these calculations, MSE curves are obtained and can be used to compare the complexity of time series. The MSE curve whose entropy values are higher for the most of time scales is considered more complex [47,53].

In this study, we used *SampEn* as an irregularity metric. In this respect, irregularity is estimated as the negative logarithm of the conditional probability that two sequences of *m* (embedding dimension) consecutive data points, which fulfil a tolerance criterion (i.e., are considered similar), still meet the criterion when their lengths are increased in one sample (Equation (6)). Therefore, the higher the self-similarity in the time-series, the lower the estimation.

Formally, given *N* data points from a time-series **X** = $\left\{x_{1},x_{2},\ldots ,x_{N}\right\}$, these steps should be followed to estimate *SampEn* [60]:

1. Form *N*-*m*+1 template vectors of *m* consecutive data points $\left\{X_{m}\left(1\right),\ldots ,X_{m}\left(N-m+1\right)\right\}$, where each template vector is defined by $X_{m}\left(i\right)$ = $\left\{x_{i},\ldots ,x_{i+m-1}\right\}$, *i* = $\left\{1,\ldots ,N-m+1\right\}$. These vectors represent *m* consecutive values of **X** commencing with the *i*-th sample.
2. Define the distance between $X_{m}\left(i\right)$ and $X_{m}\left(j\right)$, $d[X_{m}\left(i\right),X_{m}\left(j\right)]$. In this context, Chebyshev is the most common metric used to compute the distance between vectors [59],
  (2) $$d\left[X_{m}\left(i\right),X_{m}\left(j\right)\right]\;=\;max_{k=1,\ldots ,m}(|x\left(i+k-1\right)-x\left(j+k-1\right)|).$$
3. Define a tolerance criterion in terms of the standard deviation (σ) of the time series, R=r·σ, where *r* is a parameter to set. Thus, two template vectors, $X_{m}\left(i\right)$ and $X_{m}\left(j\right)$, are considered similar if their distance is less than the tolerance value:
  (3) $$d[X_{m}\left(i\right),X_{m}\left(j\right)]<R.$$
4. For each $X_{m}\left(i\right)$, count the number of vectors $X_{m}\left(j\right)$, given *i*≠*j*, that fulfil the tolerance criterion (Equation (3)). This count is denoted as $B_{i}$. Then, the frequency of patterns similar to a given one of window length *m* is calculated by
  (4) $$B_{i}^{m}\left(r\right)\;=\;\frac{B_{i}}{N-m+1},\;i\;=\;1,\ldots ,N-m+1.$$
5. Define the probability that two sequences will match for *m* points as
  (5) $$B^{m}\left(r\right)\;=\;\frac{1}{N-m}\sum_{i=1}^{N-m}B_{i}^{m}\left(r\right)$$
6. Increase the embedding dimension to *m*+1, and repeat steps 1 to 5 in order to calculate $A^{m+1}\left(r\right)$ as the probability that two vectors, $X_{m+1}\left(i\right)$ and $X_{m+1}\left(j\right)$, given *i* ≠ *j*, match for *m*+1 points.
7. Then, we can estimate *SampEn* by computing:
  (6) $$SampEn\left(m,r,N\right)\;=\;-\ln\left[\frac{A^{m+1}\left(r\right)}{B^{m}\left(r\right)}\right].$$

*SampEn* is used to estimate the MSE because, unlike approximate entropy (*ApEn*) algorithm, *SampEn* does not count self-matching, which introduces an inherent bias, and the result provided is less dependent of time series length [53]. To obtain the τ-th time-scaled version of the EEG, the original procedure was to divide the signal into *N*/τ consecutive and non-overlapping segments of length τ and average the samples of each segment [47]. Nevertheless, these calculations may cause aliasing [59]. Therefore, as Martínez-Cagigal et al. [61] proposed, we estimated time-scaled versions by decimating the original signal. For the τ-th time scale, we applied a low-pass least-squares linear-phase FIR filter to the pre-processed EEG signals (∼120 s duration) in order to reduce high frequencies. Then, we applied a downsampling procedure. That is, only every τ-th sample was kept. Finally, the irregularity of different time-scaled signals was estimated by the *SampEn* algorithm from original time series (i.e., τ = 1) up to the highest scale (τ = 20), obtaining the MSE curves for each channel by plotting the results. In this regard, we considered the scales that fulfil the Richman & Moorman criterion (i.e., $N\geq 10^{m}$) [60].

### 2.6. Statistical Analysis

The normality of the distributions of the pre- and post-training EEG measures, as well as the pre- and post-training Luria-AND scores, were explored by applying the Kolmogorov–Smirnov test. Neither EEG results (i.e., the spectral and complexity values) nor the neuropsychological scores fulfilled the parametric assumption. Thus, the non-parametric Wilcoxon signed-rank test was performed in order to assess the statistical differences between pre- and post-training EEG recordings, and analyse changes in cognitive functions. Additionally, the possible relationship between changes in RP of each frequency band and the change in EEG complexity after MI–NFT was analysed by Spearman’s rank correlation. In order to limit the number of comparisons, each MSE curve was characterised by a single value. This was obtained from the median value of the 20 time scales. We analysed each channel separately. Furthermore, to investigate the relationship between changes in MSE and neuropsychological improvements after MI–NFT, Spearman’s rank correlation was calculated separately for each Luria-AND test that showed a significant improvement. MSE curves were also characterised by estimating their median value. Finally, it is worth noting that, to overcome the problem of false discoveries due to multiple comparisons, the Benjamini–Hochberg false discovery rate (FDR–BH) correction was applied [62].

## 3. Results

### 3.1. EEG Spectral Analysis

**Figure 3:**
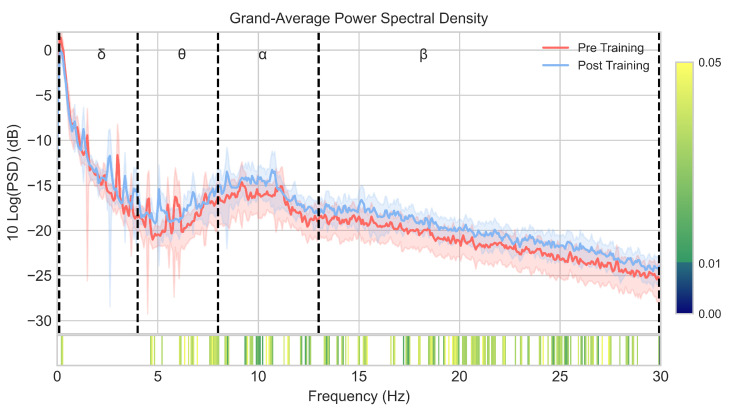
The graph depicts the grand-average of the PSD across channels and subjects. Solid lines indicate the averaged PSD for pre- (red), and post-training (blue); whereas shaded areas indicate the 95% confidence interval. Frequency bands are indicated by dashed lines (delta: δ; theta: θ; alpha: α; beta: β). Wilcoxon signed-rank test *p*-values that show significant differences (p<0.05) between pre- and post-training PSD are shown in the bottom bar. FDR–BH correction was applied.

Pre- and post-training PSDs were averaged across channels and subjects. As can be seen in Figure 3, a significant power increase (p<0.05) is found in theta, alpha, and beta frequencies, with a greater number of significant frequency bins in the alpha and beta bands. Differences between pre- and post-training RPs of each frequency band, averaged across channels and subjects, are displayed in Table 2. As shown, RP increased in theta (p<0.01), alpha (p<0.01), and beta (p<0.01) bands, while RP decreased in delta band (p<0.01), which means a power shift towards higher frequencies after NFT.

**Figure 4:**
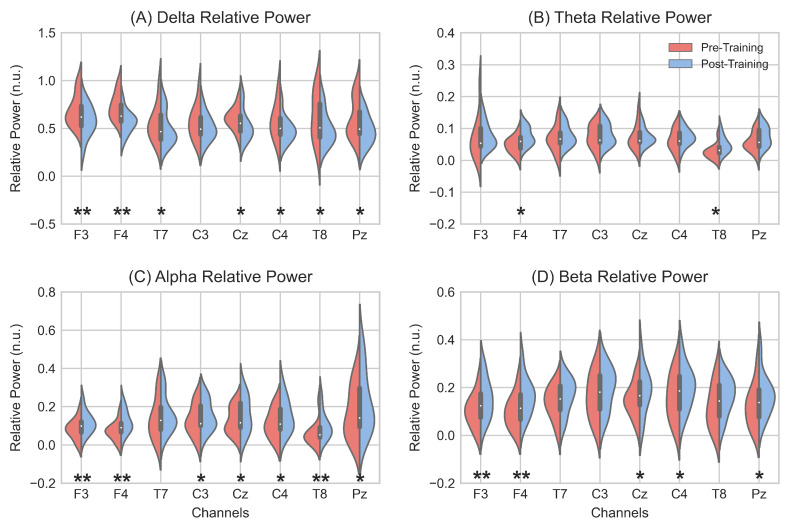
Violin plots depict pre- (red, left side) and post-training (blue, right side) RP of delta (**A**), theta (**B**), alpha (**C**), and beta (**D**) frequency bands. Significant differences are marked with * (p<0.05) and ** (p<0.01). *p*-values were corrected with FDR–BH correction.

Figure 4 depicts the intra-channel comparison between pre- and post-training RP of each frequency band. Significant changes are found in all bands, especially in delta, alpha, and beta. Frontal channels (F3 and F4) show significant differences (p<0.01) in these three bands, whilst a significant increase (p<0.05) is also found at F4 in theta band. In addition, three other channels (C4, T8, and Pz) exhibit significant RP differences (p<0.05) in three out of the four frequency bands. It is noteworthy that all eight channels present significant RP differences (p<0.05) in at least one frequency band.

### 3.2. EEG Complexity Analysis

To estimate the MSE curves, parameter values were varied in accordance with common ranges used for biomedical signals: embedding dimension *m* = $\left\{1,2\right\}$ and tolerance parameter *r* = {0.1, 0.15, 0.2, 0.25, 0.3} [60]. Hereafter, we focus our discussion on the MSE curves obtained for *m* = 2 and *r* = 0.1 (i.e., R=0.1·σ), since these values are widely used in the literature [60,63]. However, this selection of parameters did not influence the results obtained, showing their consistency across the different values. MSE curves calculated for the remaining parameter values can be found in the Supplementary Materials.

**Figure 5:**
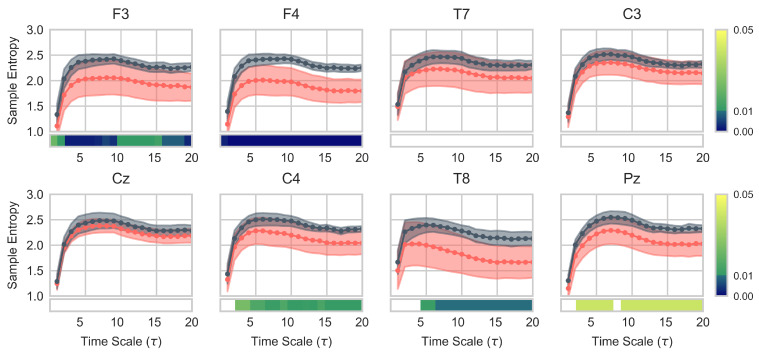
MSE values across channels (*m* = 2; *r* = 0.1). Lines with circle markers indicate pre- (red) and post-training (grey) averaged sample entropy values. Significant differences (p<0.05) for each time scale are shown in the bottom bars. *p*-values were corrected with FDR–BH correction.

MSE curves for each channel are displayed in Figure 5. Differences were evaluated for each time scale in each channel. A general increase in *SampEn* values compared to the pre-training ones is observed, except for Cz. Significant increases are found in five of the eight channels: C4 and Pz (p<0.05), and F3, F4, and T8 (p<0.01). In this regard, it is remarkable that the F3 and F4 channels show significant differences for all time scales considered, whilst significant differences are found from τ=3 onwards in C4, and from τ=5 onwards in T8. On the other hand, Pz curves show significant differences for all time scales, except for $\tau \;=\;\left\{1,2,8\right\}$.

### 3.3. Correlation Analysis of Spectral and Complexity EEG Changes

**Figure 6:**
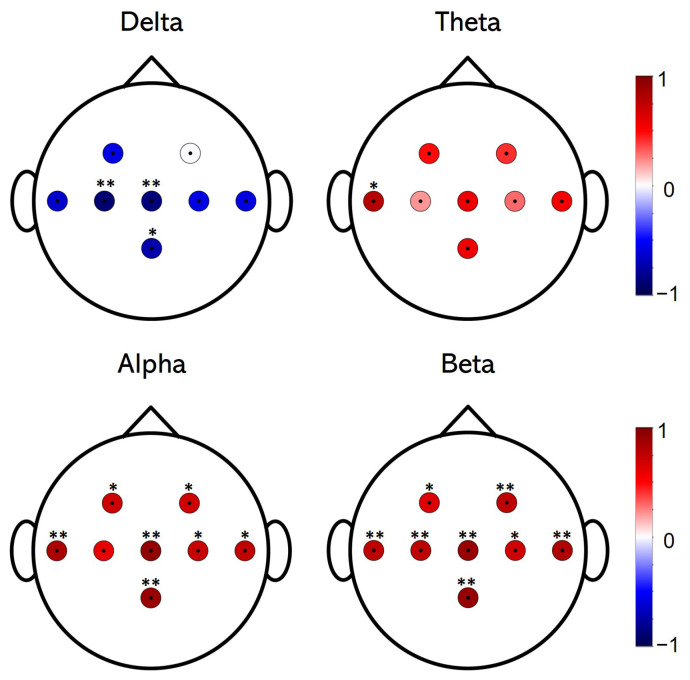
Topographic plots of Spearman’s rank correlation values between RP and MSE changes for each channel. Each frequency band under study is presented separately. Positively correlated channels are shown in red, while negatively correlated channels are shown in blue. Darker colours are related to more positively (or negatively) correlated values. Channels that revealed significant results are marked with * (*p* < 0.05) and ** (*p* < 0.01). *p*-values were corrected with FDR-BH correction.

Results obtained from Spearman’s rank correlation analysis are shown in Figure 6. A strong negative correlation is found between delta power and increased MSE. Significant results are obtained in C3 (ρ = −0.89, *p* < 0.01), Cz (ρ = −0.87, *p* < 0.01) and Pz (ρ = −0.73, *p* < 0.05), whilst negative values are also found in F3, T7, C4, and T8 (ρ < −0.58, *p* = 0.07). Moreover, the correlation analysis in the theta band shows only a significant result at T7 (ρ = 0.78, *p* < 0.05). No positive results are found in the other channels (*p* > 0.05). Regarding the analysis of the alpha band, a strong positive correlation is found in all channels, among which the most significant are T7 (ρ = 0.83, *p* < 0.01), Cz (ρ = 0.92, *p* < 0.001) and Pz (ρ = 0.90, *p* < 0.001). The rest of the channels also showed a significant result (ρ > 0.7, *p* < 0.05), with the exception of C3 (ρ = 0.6, *p* > 0.05). Finally, beta band correlation analysis yields significant and positive results for all channels. Specifically, the results obtained are (ρ = 0.64, *p* < 0.05) in F3, (ρ = 0.75, *p* < 0.01) in F4, T7, and C3, (ρ = 0.89, *p* < 0.001) in Cz, (ρ = 0.68, *p* < 0.05) in C4, (ρ = 0.81, *p* < 0.01) in T8, and (ρ = 0.9, *p* < 0.001) in Pz.

### 3.4. Luria-AND Analysis

The difference between pre- and post-training scores of Luria-AND test was analysed in order to assess cognitive changes achieved after performing NFT. In Table 3, differences between pre- and post-training Luria scores for each subject are shown, as well as results of the Wilcoxon signed rank test. As can be seen, subjects show significant improvement in the thematic draws test, conceptual activity test, and logical memory test. That is, an enhancement is found in two (intellectual and memory functions) of the five cognitive functions assessed by Luria-AND test. The scores of the three Luria-AND tests that showed a significant improvement were used to analyse the possible correlation between cognitive improvement and increased EEG complexity after MI–NFT. Correlation values were calculated for each of the eight channels used. However, no significant results (*p* > 0.05) were extracted from the analysis.

## 4. Discussion

In this study, we employed a dataset composed of resting state EEG signals from 11 healthy elderly subjects in order to comprehensively explore the EEG changes induced by an MI–NFT aimed at cognitive enhancement. To this end, we applied linear and non-linear analyses. In this regard, spectral and complexity analyses allowed us to quantitatively assess the EEG changes from a broad point of view. Furthermore, to our knowledge, this is the first study that measured both spectral and complexity EEG changes induced by an MI–NFT in the elderly, as well as the correlation between these induced changes. The increase found in the post-training MSE values supports our hypothesis that MI–NFT can induce changes in EEG complexity and proves the effectiveness of MSE as a robust technique for assessing NFT outcomes in future studies. Moreover, the results of the Luria-AND test showed an improvement in the intellectual and memory abilities of older people after MI–NFT. This reinforces the idea that MI–NFT may be a suitable approach to help the elderly counteract the effects of age-related cognitive decline.

### 4.1. Induced EEG Spectral Changes

In the present study, different spectral analyses were applied to assess EEG changes in the frequency domain. Regarding trained bands, we observed a general increase in the power of alpha (8–13 Hz) and beta (13–30 Hz) bands in the grand-average PSD analysis (Figure 3). Concerning RP analysis (Table 2), the averaged power of both frequency bands showed a significant increase (p<0.01) after NFT. Therefore, our results suggest the ability of aging brains to voluntarily regulate alpha and beta bands with the help of an MI-based BCI tool. This is in line with reports of previous classical NFT studies. For instance, Staufenbiel et al. [25] proposed a beta-enhancing NFT protocol. Although significant differences in the beta band were not achieved at post-training resting EEG measures, an increase in beta power was found at the end of the sessions compared to the power at baseline, suggesting the trainability of the beta band in the elderly. Moreover, Marlats et al. [23] studied the ability of elderly patients with mild cognitive impairment (MCI) to stimulate their SMR, finding an increase in theta and alpha band powers after NFT. On the other hand, it is worth noting that the increase we found in the power of trained bands was achieved with only five MI–NFT sessions, with an average duration of 90 min, which suggests that little training is necessary for older people to be able to gain control over their brain activity by performing MI. With regard to RP changes across channels (Figure 4), we found a significant increase in seven out of the eight channels in alpha band and in five of the eight channels in beta band. The main increase was found in frontal channels (F3 and F4). Increases in alpha and beta power in the frontal region after NFT have been previously reported. Becerra et al. [24] proposed an NFT protocol based on the reduction in abnormally high absolute theta power in an elderly healthy population. As a result of this training, subjects exhibited a significant improvement in the RP of alpha band in frontal, right-temporal, and occipital regions. Campos da Paz et al. [22] conducted an NFT protocol in elderly patients based on SMR. An enhancement of the power was found in theta and beta bands in the frontal region after NFT. Interestingly, the frontal region has been associated with cognitive functions, such as working memory, attention, fluid intelligence, or language comprehension [38,64,65,66,67]. Therefore, the significant increase observed in frontal region in this study, as well as in the aforementioned ones, may reflect a change in EEG patterns, which could lead to an improvement in these related cognitive functions.

Regarding EEG changes in the slow frequency bands, an overall decrease in the delta band power was found, as can be seen in Table 2. Thus, taking into account the increase in alpha and beta bands, a power shift towards rapid frequencies was achieved after the MI–NFT. Considering the EEG slowing process due to aging [11,12,14], it is suggested that an MI–NFT may be helpful for older people to counteract the progression of this phenomenon. In the intra-channel analysis (Figure 4), a significant decrease was revealed in all channels except for C3. The main decrease (p<0.01) was observed in frontal channels. This, in view of the significant increase in alpha and beta power found in the same channels, suggests a major effect of MI–NFT on the frontal region. On the other hand, the theta band power was found to be increased after the MI–NFT (Table 2). This can be seen in the power spectral distribution (Figure 3), where certain frequencies showed a significant increase in power. However, the enhancement in theta power was less remarkable in comparison to alpha and beta bands, as can be observed in the intra-channel analysis (Figure 4). Our results are in line with the aforementioned study conducted by Marlats et al. [23]. They reported an increase in the power of the theta band, although their protocol was based on SMR enhancement and theta suppression. They suggested that the increase in the theta band power may come from the decrease observed in delta power. This can be interpreted as an improvement due to the faster brain signals after NFT [23]. Even though an abnormal increase in the power of the theta band has been linked to age-related cognitive decline [11,12,14], frontal theta activity has also been reported to play a key role in the control of some cognitive functions, especially working memory [19,68,69]. For this reason, several studies have proposed to enhance this frequency band in their NFT protocols [17,18,19]. In this sense, the need to up-regulate theta band (i.e., to achieve cognitive enhancement) or, conversely, to down-regulate it (i.e., to counteract the power shift caused by aging) is still uncertain.

Finally, it is interesting to note that the MI–NFT influenced delta and theta power, although these bands remained untrained. In fact, previous NFT studies have reported non-specific frequency changes in flanking frequency bands [18,23,70], supporting the proposed interdependence between frequency bands [71]. Consequently, as suggested by Jurewicz et al. [70], the effects in the untrained bands can be interpreted as a response to the change achieved in the trained bands. This side effect could be aimed at balancing the spectral power distribution of brain activity after training.

### 4.2. Induced EEG Complexity Changes

The results obtained from MSE curves (Figure 5) showed a general increase of *SampEn* values (i.e., irregularity) at most time scales for seven out of the eight channels. According to Costa et al. [53], the increase in irregularity values found at most time scales implies a greater complexity of the post-training EEG signal. Hence, our findings in MSE analysis support our hypothesis that MI–NFT is able to influence the brain signals of the elderly. We consider this result to be relevant, as it reinforces the idea that the MI–NFT paradigm can be a suitable approach for cognitive training studies based on neurofeedback. In addition, it has been shown that complexity-based measures can characterise these EEG changes. On the other hand, it is interesting to note that the increase in EEG complexity was achieved with a reduced number of training sessions, suggesting that changes in brain signal complexity can be induced with few MI–NFT sessions. It is also noteworthy that the significant increase in EEG complexity was obtained for all combinations of parameter values, as can be seen in the Supplementary Materials. This demonstrates the robustness of the MSE as a complexity-based evaluation metric.

Previously, the MSE analysis has been applied in the estimation of EEG complexity in AD and MCI patients [48,49,51,72]. These studies showed a lower complexity of the resting state EEG signal in AD patients compared to control subjects. Their results are consistent with the hypothesis that AD is characterised by a reduction of complexity [73], which has been suggested to reflect a decrease in non-linear neural dynamics caused by a loss of connectivity of local neural networks and/or neuronal death [12]. Moreover, MSE analysis has been applied to explore the changes in EEG complexity throughout aging, showing a prominent increase in EEG complexity from childhood to adolescence [74], and a lower EEG complexity in elderly subjects compared to young ones [50]. Thus, in view of the likely association between a reduced EEG complexity and the cognitive decline process, the increase observed in our study post-MI–NFT may lead to a possible alleviation of the effects of age-related cognitive decline. Furthermore, the most significant differences were found in frontal channels (Figure 5). A similar result was also reported by Van Noordt et al. [74], showing a main increase in EEG complexity located in fronto-central region. They suggest that the increase observed may reflect a maturational change in underlying brain networks.

Finally, although non-linear analysis is widely used in the characterisation of EEG changes throughout the process of cognitive decline [48,49,50,51,72], to the best of our knowledge, MSE has not been generally considered as an evaluation metric in previous NFT studies. That is, few NFT studies have applied MSE measurement in order to find NFT-induced EEG changes [52,75]. In fact, to our knowledge, there is only one study that reported the influence of classical NFT on EEG complexity in the elderly [52]. In this study, an increase in EEG complexity located in fronto-central region was shown. Therefore, based on these results and those obtained in our study, we propose the use of MSE to assess NFT outcomes, because it can supplement the information provided by linear methods. Thus, combining spectral analysis with complexity-based analysis methods may help to further develop the field of NFT by suggesting new ways to compare NFT results from different studies.

### 4.3. Correlations between Spectral Power and EEG Complexity

The results obtained from the correlation analyses showed a significant correlation between the increase in EEG complexity and the change in the RP of the frequency bands after MI–NF. This correlation is notably prominent in delta, alpha, and beta bands. In the theta analysis, only one significant result was obtained, in the T7 channel. This agrees with the results obtained in the analysis of the RP change of each frequency band in each channel (Figure 3). Therefore, these results suggest that the overall increase in EEG complexity observed after MI–NFT is correlated with a homogeneous decrease in delta RP, as well as an increase in alpha and beta RP. More specifically, with regard to frontal channels, which showed the most prominent changes in both spectral and complexity analyses, significant positive results were found in alpha and beta correlation analyses. This suggests that the significant increase in complexity observed in these channels is correlated with the increase in the RP of the trained bands by the MI–NFT paradigm. Therefore, changes observed in these EEG features could be seen as evidence of the same phenomenon: an enhancement of brain signal regulation induced by the MI–NFT. To our knowledge, correlation between spectral and complexity EEG changes after MI–NFT have never been studied before. In view of the results of previous studies, in which alpha and beta band activities were related to different cognitive functions [19,38,39,40,41,42,43,44,45,46], as well as the aforementioned connection between EEG complexity and cognitive state [48,49,50,51,72], it seems interesting to further investigate these findings. Thus, this may help to better characterise the results of future NFT studies, as well as provide a deeper insight into the NFT-induced EEG changes.

### 4.4. Changes in Neuropsychological Functions

The Luria-AND test was performed twice to study the possible influence of MI-based NFT on the cognitive functions of the subjects. After a statistical analysis, significant improvements were found in intellectual (i.e., thematic draws and immediate conceptual activity tests) and memory functions (i.e., logical memory test). Thus, according to these results, an MI-based NFT may be helpful to improve cognitive functions in elderly people, such as those related to memory and intelligence. However, the correlation analysis of cognitive enhancement and increased EEG complexity did not establish a significant relationship between the two changes observed. The absence of significant results may be due to the limited sample size available, as well as the high variability in the changes of the Luria-AND scores. Despite this, our results highlight the importance of further study of the relationship between EEG changes in the frontal region and improvements in memory and intellectual functions. In fact, previous neuropsychological and neuroimaging studies have described the role played by frontal region in human memory and intelligence [38,64,65,66,67]. Furthermore, it has been suggested to be a correlation between the performance of intelligence and memory-related functions in frontal cortex [67]. Therefore, the fact that both spectral and complexity analysis showed a more prominent change in EEG features in frontal channels encourages further research into its connection with the cognitive improvements observed in subjects after MI–NFT sessions.

### 4.5. Limitations and Future Research Lines

Even though our results showed improvements in the subjects’ brain signals and in certain cognitive functions, there are several limitations that must be taken into account. Firstly, the study was conducted using a dataset composed of recordings from 11 subjects and their scores from neuropsychological tests. Hence, our results should be interpreted with caution, as it would be desirable to extend the sample size. However, it is also necessary to highlight the existence of other studies in the literature that were carried out with a similar number of subjects. For instance, Angelakis et al. [76] carried out their study with three subjects in the experimental group and 3 subjects in the control group. Becerra et al. [24] conducted a study with seven subjects in the experimental group and seven in the control group. Staufenbiel et al. [25] published a study in which two training protocols were proposed, each of which was applied to 10 people, with no control group. Campos da Paz et al. [22] published a study with 17 healthy subjects and without a control group. This illustrates the difficulty of conducting NFT studies with a large sample size. In this regard, not only must the process of collecting participants be taken into account, but so should the limited time available for the studies and the logistical complications that may arise. This is why, despite the small sample size used in the aforementioned studies, their results constitute a knowledge base on which to build future studies of cognitive training based on NFT. Therefore, we hope that the results we report will encourage future studies to further investigate the influence of the MI–NFT paradigm on EEG features and the neuropsychological state of users. Additionally, we were not able to establish a significant correlation between increased EEG complexity and observed cognitive improvement in memory and intellect functions. Therefore, further MI–NFT studies are needed in the future to shed light on the possible relationship between EEG complexity in frontal channels and the enhancement of cognitive functions such as memory or intelligence. Therefore, MSE could be established as a suitable and helpful measure for the evaluation of NFT studies. It is worth noting the need to further study the correlation between changes in RP and changes in MSE values after MI–NFT. In addition, the influence of trained frequency bands on untrained ones needs to be studied in depth in order to shed light on the issue. A clearer understanding of this phenomenon would help to design better NFT protocols.

Secondly, we analysed the effects of the MI–NFT paradigm in cognitive training. Although this training paradigm has shown promising results in stroke rehabilitation studies [3,4,31,32,33,34,35], it has not yet been explored in depth in cognitive training studies based on neurofeedback techniques. Since the MI–NFT paradigm allows for the development of more engaging [32,37] and complex training tasks, as presented in Section 2.1, it is interesting to further investigate the influence of the MI–NFT paradigm on the cognitive state of users. It would also be desirable to conduct studies comparing the effectiveness of the classical and MI–NFT training paradigms, as well as their influence on the motivation of users of the training.

Finally, the inclusion of a placebo control group under study in future studies, as recommended in [77], would help to distinguish both EEG changes and improvements in neuropsychological tests induced by real MI–NFT from those due to non-specific effects. Furthermore, the assessment of changes in cognitive functions was carried out immediately after the end of the training. Nevertheless, since the aim of NFT is to lead to physiological changes that reduce the effects of neural disorders [5], it would be desirable to perform a longitudinal study of cognitive status of patients some time after the end of the training. For instance, an MI–NFT study, aimed at restoring the neurological functions of stroke patients, showed sustained improvements 6 months after the MI–NFT-based therapy ended [4]. Hence, by performing longitudinal studies, we could gain a better understanding of the benefits of MI–NFT for the elderly.

## 5. Conclusions

RP and MSE changes in the EEG signal of 11 healthy elderly subjects after five sessions of MI–NFT using a BCI system were investigated. Our results suggest that MI–NFT is a suitable approach to induce changes in brain activity in the elderly, and furthermore, these changes are not only shown as a power shift towards rapid frequencies, but also as an increase in the complexity of the EEG signal. In fact, we have shown a correlation between spectral changes and increased EEG complexity induced by MI–NFT. We can conclude that a complexity analysis based on MSE is convenient for characterising the effects of MI–NFT. Therefore, combining spectral analyses with non-linear methods may be useful in future studies, as it provides a new perspective on the assessment of the MI–NFT effects and may be helpful to reach a deeper understanding of how neurofeedback affects the brain after training. Furthermore, the analysis of scores from neuropsychological tests showed an improvement in intellectual and memory functions in elderly people after MI-based NFT. Accordingly, MI–NFT may help the elderly counteract the effects of age-related cognitive decline.

## Figures and Tables

**Table 1:** Socio-demographic data of the population included in the study.

Socio-Demographic Data		
**Identifier**	**Sex**	**Age (Years)**
U01	Female	70
U02	Female	65
U03	Female	65
U04	Male	71
U05	Male	68
U06	Female	73
U07	Male	81
U08	Male	65
U09	Female	75
U10	Female	70
U11	Female	60

**Table 2:** Pre- and post-training RP values of each frequency band, averaged across channels, and its standard deviation (std). The Wilcoxon signed-rank test *p*-values with FDR-BH correction are shown in the last column.

	Delta	Theta	Alpha	Beta
Pre-NFT RP (mean ± std)	0.622 ± 0.154	0.060 ± 0.029	0.112 ± 0.068	0.132 ± 0.073
Post-NFT RP (mean ± std)	0.520 ± 0.131	0.073 ± 0.028	0.149 ± 0.077	0.175 ± 0.080
*p*-value	0.0013	0.0013	0.0013	0.0034

**Table 3:** Differences between pre- and post-training Luria scores for each subject. Test score range is 0–90. Results from statistical analysis of changes in Luria-AND scores are shown in the first column from the right. *p*-values were corrected with FDR–BH. Significant values (p<0.05) are in bold.

Test	U01	U02	U3	U4	U5	U6	U7	U8	U9	U10	U11	Mean ± Std	*p*-Value
Attentional control	0	0	5	−15	55	5	0	45	−5	5	0	8.6 ± 21.3	0.1310
Thematic draws	5	0	0	5	0	25	10	15	0	0	15	6.8 ± 8.4	**0.0403**
Conceptual activity	10	5	5	0	0	10	5	5	0	10	0	4.5 ± 4.2	**0.0403**
Immediate memory	−5	0	0	20	10	10	20	0	0	−10	0	4.1 ± 9.7	0.1074
Logical memory	−5	0	0	30	15	20	25	−5	15	10	20	11.4 ± 12.3	**0.0403**
Receptive speech	5	0	5	15	0	5	0	10	−10	5	10	4.1 ± 6.6	0.0655
Expressive speech	0	0	10	15	0	10	−15	10	10	5	0	4.1 ± 8.3	0.1102
Visual perception	5	0	25	30	35	20	−10	20	−10	5	0	10.1 ± 15.8	0.0590
Spatial orientation	0	−10	15	10	0	−5	15	15	15	−5	35	7.7 ± 13.1	0.0590

## Data Availability

The dataset analysed in the study is available from the authors upon reasonable request.

## Supplementary Materials

The following are available online at https://www.mdpi.com/1099-4300/23/12/1574/s1, Figure S1: MSE values across channels (*m* = 1; *r* = 0.1). Lines with circle markers indicate pre- (red) and post-training (grey) averaged sample entropy values. Significant differences (p<0.05) for each time scale are shown in the bottom bars. *P*-values were corrected with FDR-BH correction. Figure S2: MSE values across channels (*m* = 1; *r* = 0.15). Lines with circle markers indicate pre- (red) and post-training (grey) averaged sample entropy values. Significant differences (p<0.05) for each time scale are shown in the bottom bars. *P*-values were corrected with FDR-BH correction. Figure S3: MSE values across channels (*m* = 1; *r* = 0.2). Lines with circle markers indicate pre- (red) and post-training (grey) averaged sample entropy values. Significant differences (p<0.05) for each time scale are shown in the bottom bars. *P*-values were corrected with FDR-BH correction. Figure S4: MSE values across channels (*m* = 1; *r* = 0.25). Lines with circle markers indicate pre- (red) and post-training (grey) averaged sample entropy values. Significant differences (p<0.05) for each time scale are shown in the bottom bars. *P*-values were corrected with FDR-BH correction. Figure S5: MSE values across channels (*m* = 1; *r* = 0.3). Lines with circle markers indicate pre- (red) and post-training (grey) averaged sample entropy values. Significant differences (p<0.05) for each time scale are shown in the bottom bars. *P*-values were corrected with FDR-BH correction. Figure S6: MSE values across channels (*m* = 2; *r* = 0.1). Lines with circle markers indicate pre- (red) and post-training (grey) averaged sample entropy values. Significant differences (p<0.05) for each time scale are shown in the bottom bars. *P*-values were corrected with FDR-BH correction. Figure S7: MSE values across channels (*m* = 2; *r* = 0.15). Lines with circle markers indicate pre- (red) and post-training (grey) averaged sample entropy values. Significant differences (p<0.05) for each time scale are shown in the bottom bars. *P*-values were corrected with FDR-BH correction. Figure S8: MSE values across channels (*m* = 2; *r* = 0.2). Lines with circle markers indicate pre- (red) and post-training (grey) averaged sample entropy values. Significant differences (p<0.05) for each time scale are shown in the bottom bars. *P*-values were corrected with FDR-BH correction. Figure S9: MSE values across channels (*m* = 2; *r* = 0.25). Lines with circle markers indicate pre- (red) and post-training (grey) averaged sample entropy values. Significant differences (p<0.05) for each time scale are shown in the bottom bars. *P*-values were corrected with FDR-BH correction. Figure S10: MSE values across channels (*m* = 2; *r* = 0.3). Lines with circle markers indicate pre- (red) and post-training (grey) averaged sample entropy values. Significant differences (p<0.05) for each time scale are shown in the bottom bars. *P*-values were corrected with FDR-BH correction.
