# Supplementary material for: Neurofeedback Training Based on Motor Imagery Strategies Increases EEG Complexity in Elderly Population
Source: Entropy (Basel). 2021 Nov 25;23(12):1574. doi: 10.3390/e23121574 (PMC8700498; doi:10.3390/e23121574)
Supplement: Supplementary file 1 [file entropy-23-01574-s001.zip › supplementary_material.pdf]

Supplementary Materials: Neurofeedback Training Based on Motor Imagery Strategies Increases EEG Complexity in Elderly Population

Diego Marcos-Martínez ,Víctor Martínez-Cagigal , Eduardo Santamaría-Vázquez , Sergio Pérez-Velasco and Roberto Hornero

For the sake of completeness, MSE curves obtained for the different parameter values are shown below. The values considered were: embedding dimension $m = \{1,2\}$; tolerance parameter $r = \{0.1, 0.15, 0.2, 0.25, 0.3\}$.

1. Embedding Dimmension $m = 1$

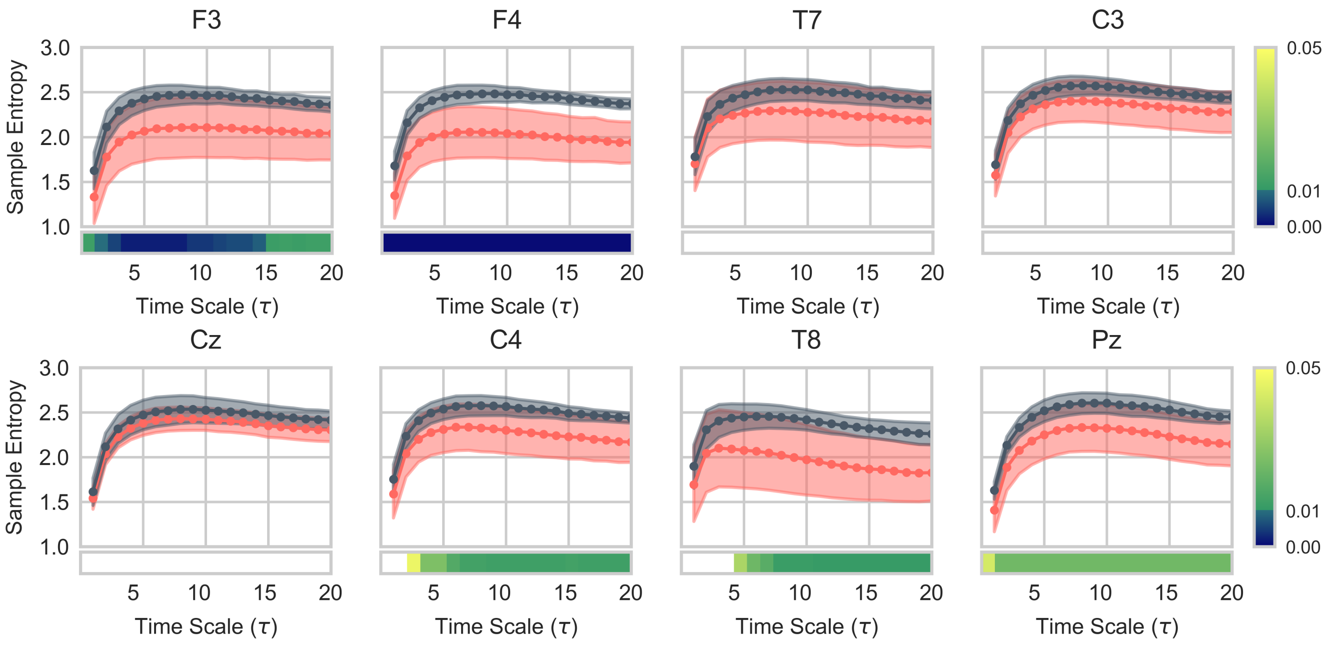

Figure S1. MSE values across channels ($m = 1$; $r = 0.1$). Lines with circle markers indicate pre- (red) and post-training (grey) averaged sample entropy values. Significant differences ($p < 0.05$) for each time scale are shown in the bottom bars. *P*-values were corrected with FDR-BH correction.

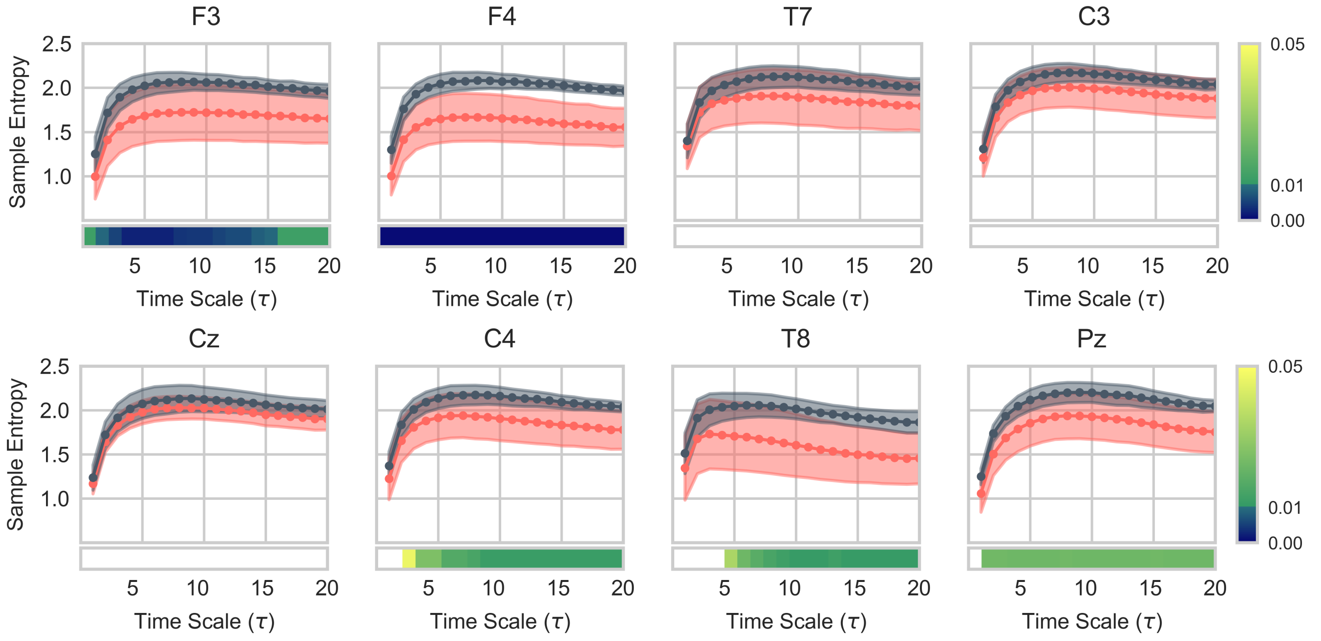

Figure S2. MSE values across channels (m = 1; r = 0.15). Lines with circle markers indicate pre- (red) and post-training (grey) averaged sample entropy values. Significant differences ($p < 0.05$) for each time scale are shown in the bottom bars. P-values were corrected with FDR-BH correction.

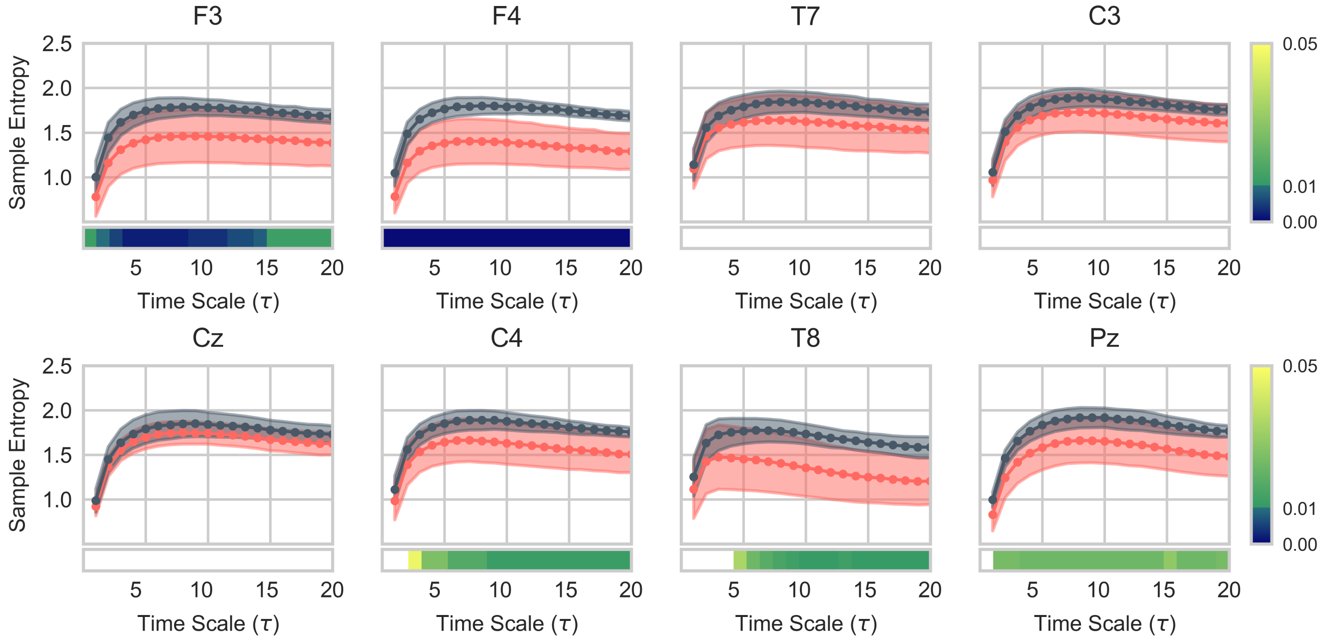

Figure S3. MSE values across channels (m = 1; r = 0.2). Lines with circle markers indicate pre- (red) and post-training (grey) averaged sample entropy values. Significant differences ($p < 0.05$) for each time scale are shown in the bottom bars. P-values were corrected with FDR-BH correction.

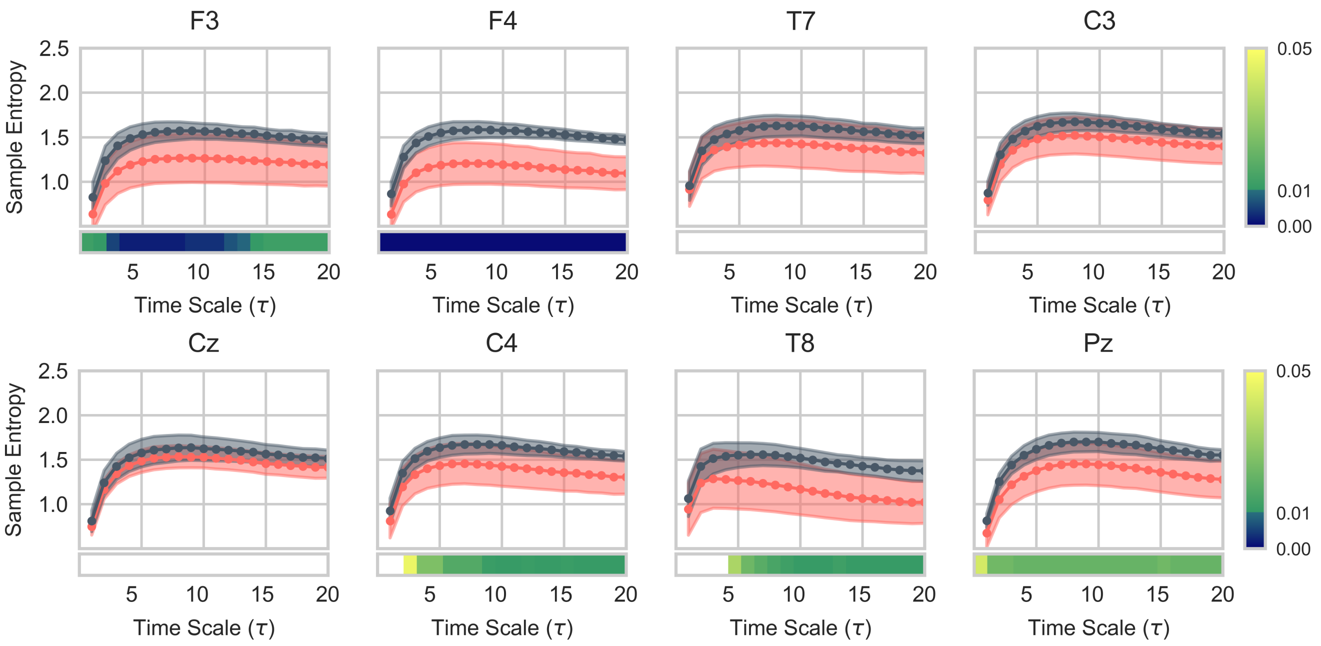

Figure S4. MSE values across channels (m = 1; r = 0.25). Lines with circle markers indicate pre- (red) and post-training (grey) averaged sample entropy values. Significant differences ($p < 0.05$) for each time scale are shown in the bottom bars. *P*-values were corrected with FDR-BH correction.

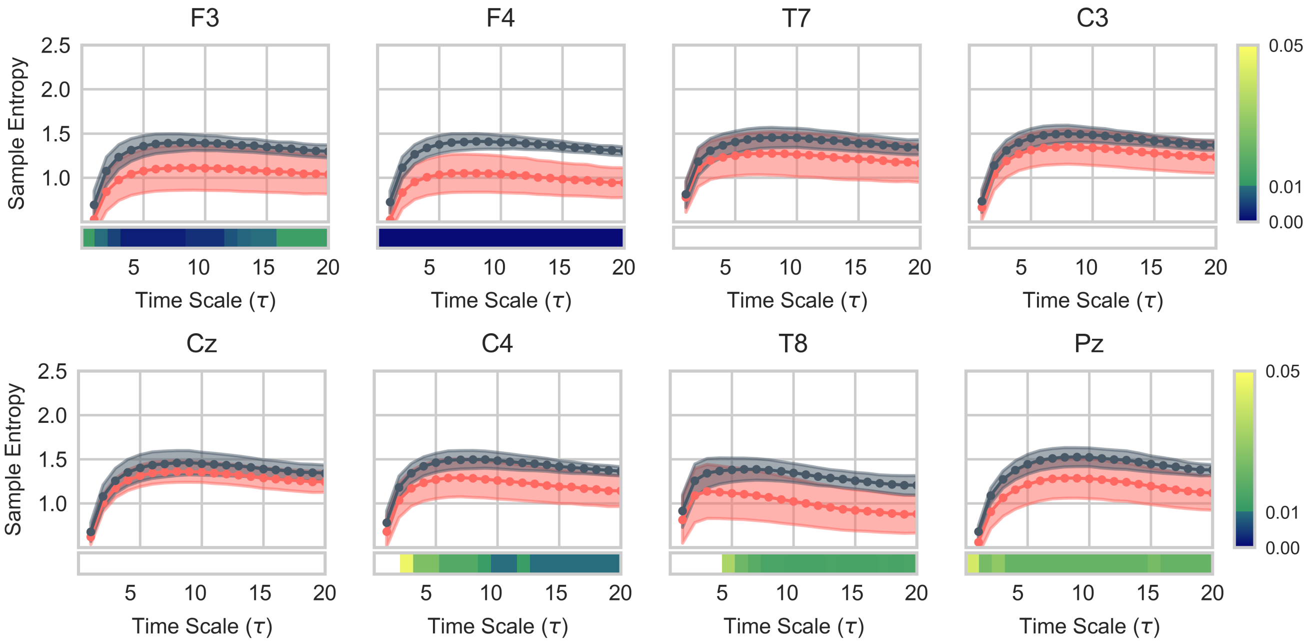

Figure S5. MSE values across channels (m = 1; r = 0.3). Lines with circle markers indicate pre- (red) and post-training (grey) averaged sample entropy values. Significant differences ($p < 0.05$) for each time scale are shown in the bottom bars. *P*-values were corrected with FDR-BH correction.

2. Embedding Dimmension $m = 2$

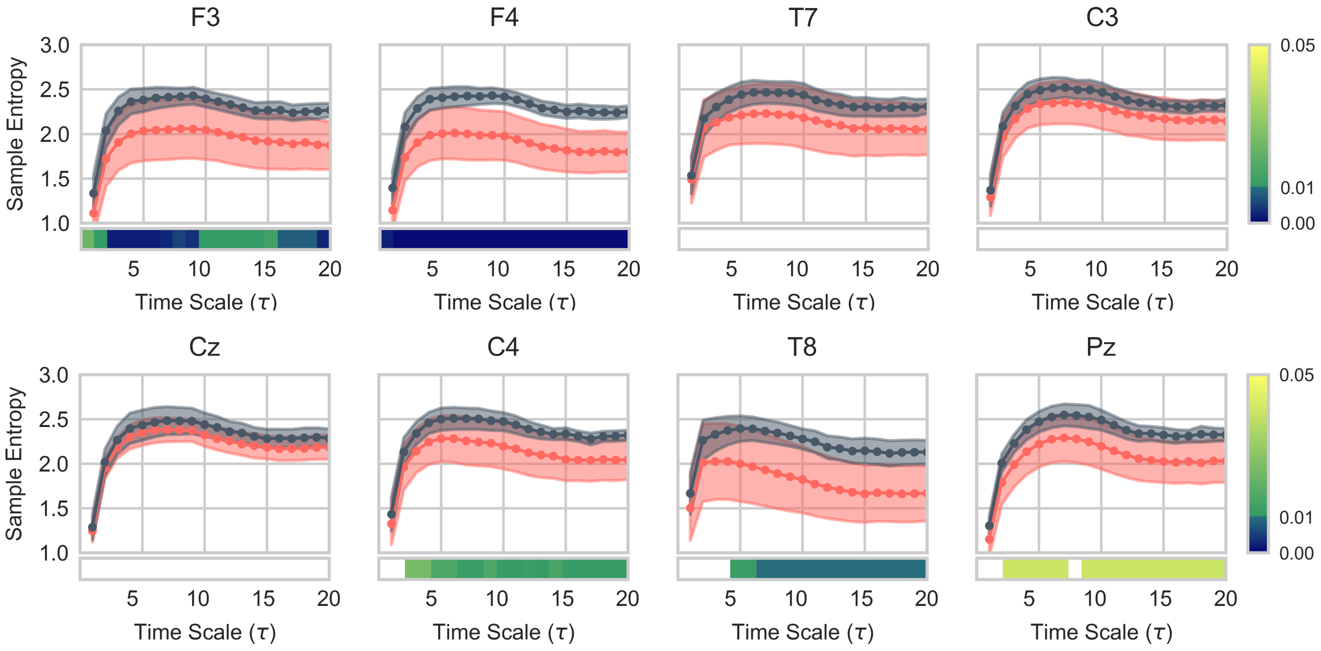

Figure S6. MSE values across channels ($m = 2$; $r = 0.1$). Lines with circle markers indicate pre- (red) and post-training (grey) averaged sample entropy values. Significant differences ($p < 0.05$) for each time scale are shown in the bottom bars. *P*-values were corrected with FDR-BH correction.

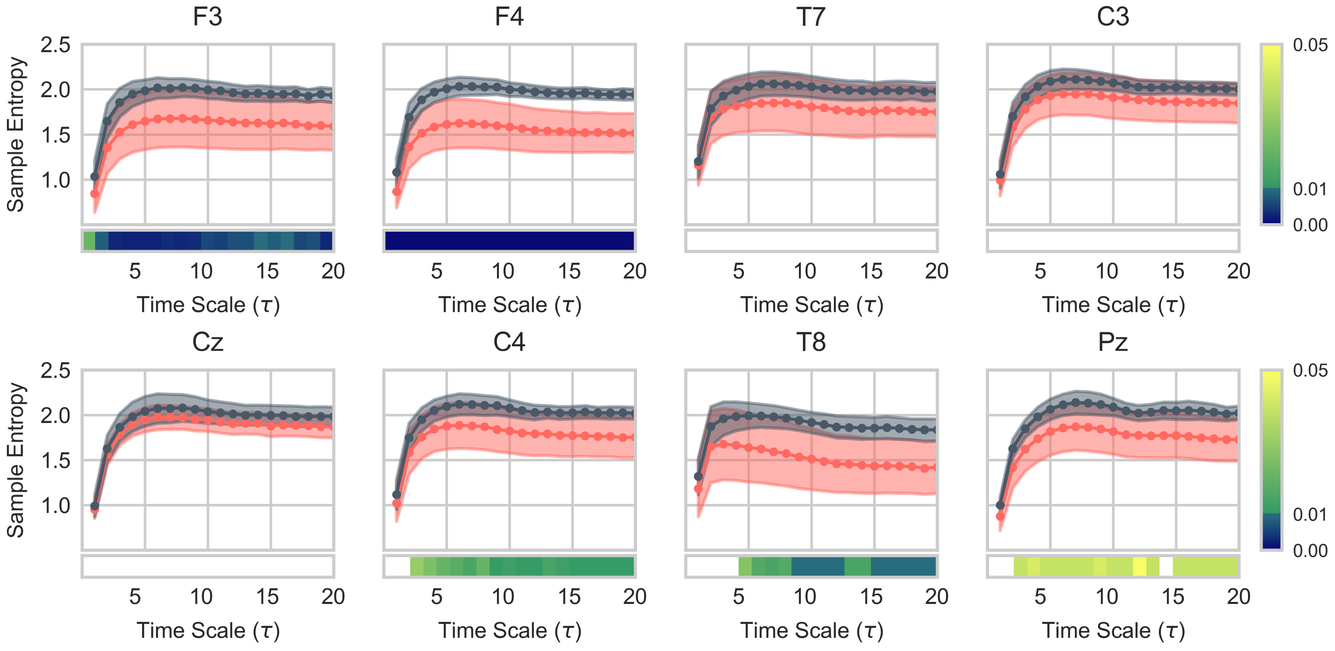

Figure S7. MSE values across channels ($m = 2$; $r = 0.15$). Lines with circle markers indicate pre- (red) and post-training (grey) averaged sample entropy values. Significant differences ($p < 0.05$) for each time scale are shown in the bottom bars. *P*-values were corrected with FDR-BH correction.

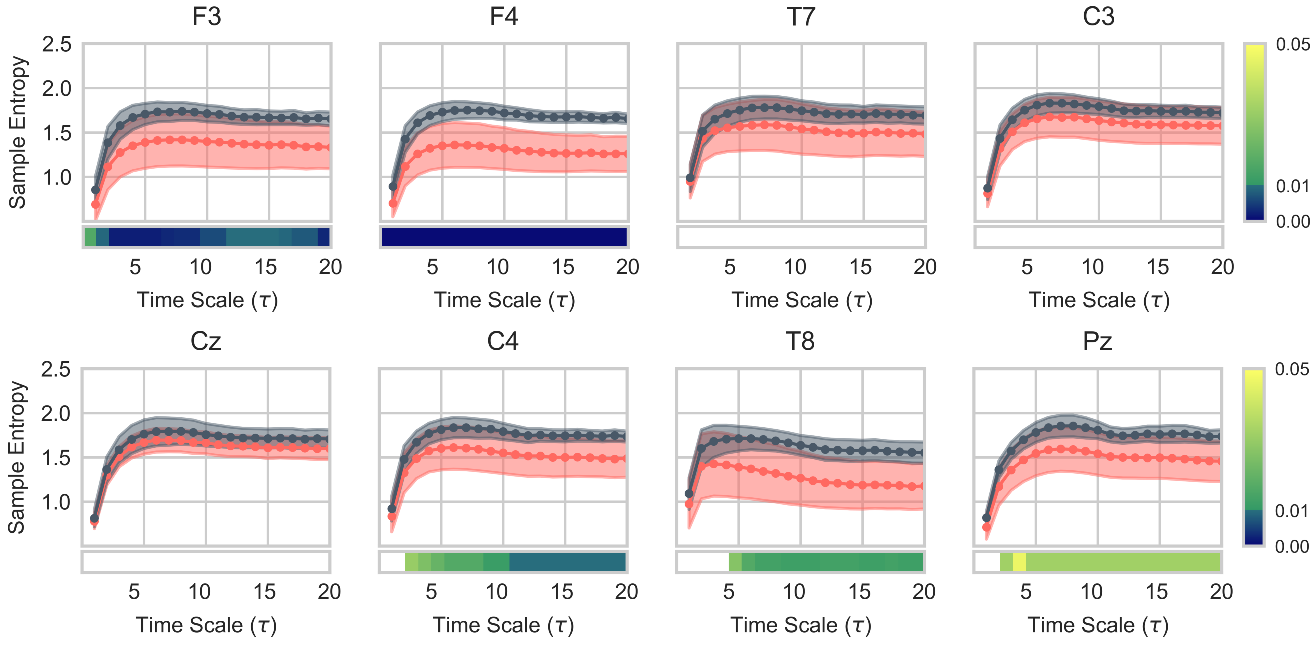

Figure S8. MSE values across channels (m = 2; r = 0.2). Lines with circle markers indicate pre- (red) and post-training (grey) averaged sample entropy values. Significant differences ($p < 0.05$) for each time scale are shown in the bottom bars. P-values were corrected with FDR-BH correction.

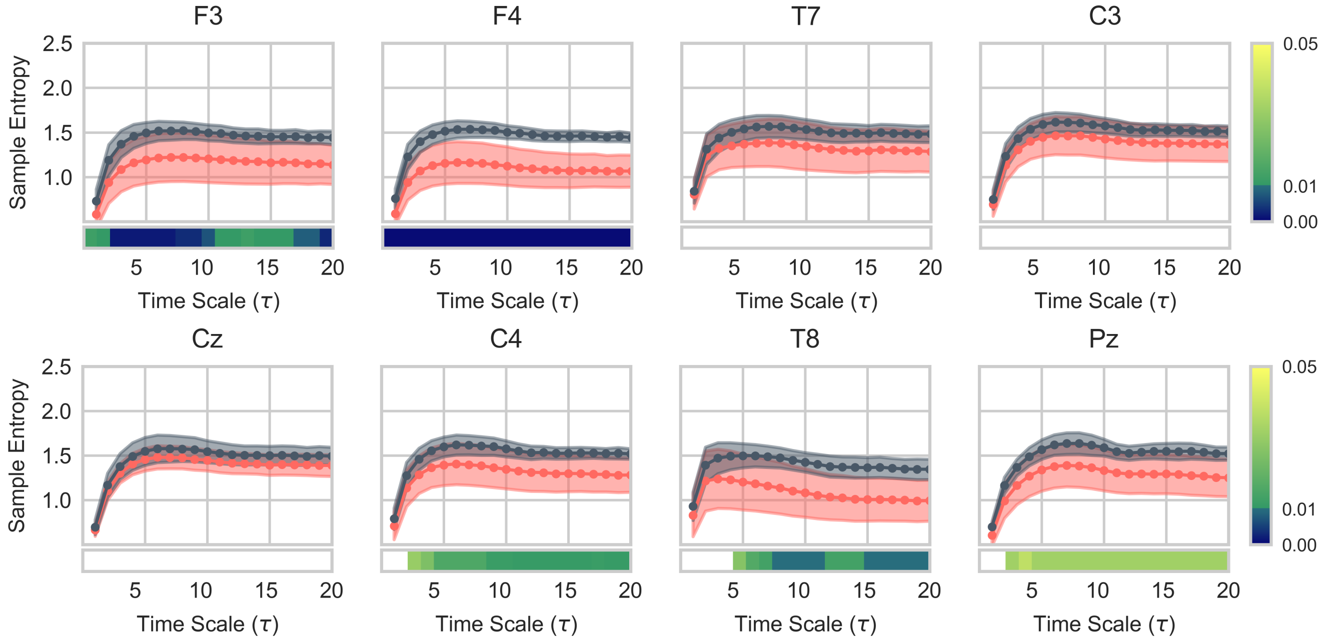

Figure S9. MSE values across channels (m = 2; r = 0.25). Lines with circle markers indicate pre- (red) and post-training (grey) averaged sample entropy values. Significant differences ($p < 0.05$) for each time scale are shown in the bottom bars. P-values were corrected with FDR-BH correction.

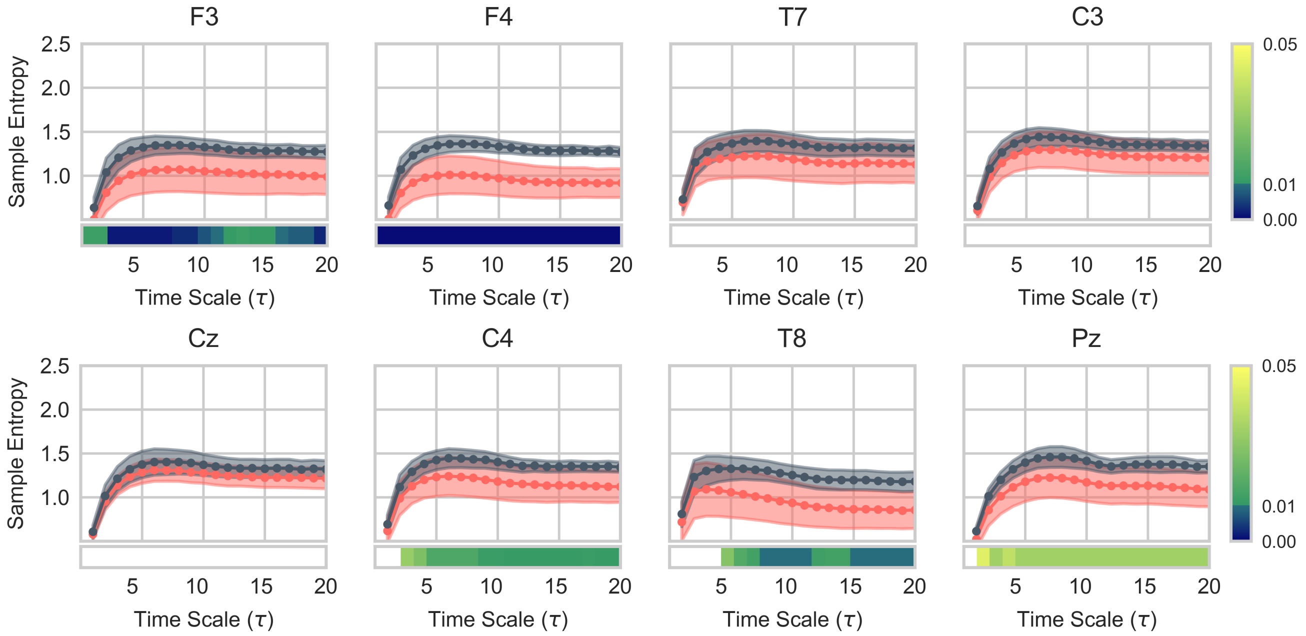

Figure S10. MSE values across channels (m = 2; r = 0.3). Lines with circle markers indicate pre- (red) and post-training (grey) averaged sample entropy values. Significant differences ($p < 0.05$) for each time scale are shown in the bottom bars. P-values were corrected with FDR-BH correction.
